# An evaluation of financial losses due to lumpy skin disease outbreaks in dairy farms of northern Thailand

**DOI:** 10.3389/fvets.2024.1501460

**Published:** 2025-01-24

**Authors:** Wittawat Modethed, Khwanchai Kreausukon, Tawatchai Singhla, Kittikorn Boonsri, Kidsadagon Pringproa, Nattawooti Sthitmatee, Paramintra Vinitchaikul, Supitchaya Srisawang, Roderick Salvador, Simon Gubbins, Georgina Limon, Veerasak Punyapornwithaya

**Affiliations:** ^1^Faculty of Veterinary Medicine, Chiang Mai University, Chiang Mai, Thailand; ^2^Laboratory of Veterinary Vaccine and Biological Products, Faculty of Veterinary Medicine, Chiang Mai University, Chiang Mai, Thailand; ^3^Research Center of Veterinary Biosciences and Veterinary Public Health, Chiang Mai University, Chiang Mai, Thailand; ^4^College of Veterinary Science and Medicine, Central Luzon State University, Science City of Muñoz, Nueva Ecija, Philippines; ^5^The Pirbright Institute, Pirbright, United Kingdom

**Keywords:** financial losses, lumpy skin disease, outbreaks, dairy farms, Thailand

## Abstract

Lumpy skin disease (LSD) poses a significant threat to the cattle industry, resulting in adverse economic consequences in affected countries. This study aims to estimate the financial losses due to LSD outbreaks in dairy farms in northern Thailand. Based on a retrospective study, data was collected using a standardized questionnaire from 100 farms affected by LSD outbreaks (outbreak farms) and 33 farms that did not experience LSD outbreaks (non-LSD outbreak farms) in two dairy farming areas that experienced LSD outbreaks between June and December 2021. In outbreak farms, the average total financial losses was 727.38 USD per farm, significantly higher than the 349.19 USD per farm observed in non-LSD outbreak farms. The primary cause of financial loss in outbreak farms was mortality. Reductions in milk sold due to a drop in milk production, and the need to discard milk because of the withdrawal time of antibiotics used for treating secondary infections on affected cattle, also contributed substantially to the financial losses. On farms without LSD outbreaks, the main expenses were related to vaccination and disease prevention, amounting to 130.66 USD and 218.53 USD per farm, respectively. LSD outbreaks negatively affect all farms in the outbreak areas, as both outbreak farms and non-LSD outbreak farms had to bear prevention costs. In the post-outbreak phase, the primary activities focused on continued monitoring of new LSD cases and conducting surveillance, carried out collaboratively by farmers and livestock authorities. This is the first study in Thailand providing valuable insights into the financial implications of LSD outbreaks for farmers, highlighting the substantial financial consequences of the disease. The findings from this study are beneficial for decision making, efficient resource allocation and the development of effective mitigation strategies.

## 1 Introduction

Lumpy skin disease (LSD) is recognized as a notifiable transboundary animal disease, mainly affecting cattle. The causative agent of LSD is lumpy skin disease virus (LSDV), a member of the *Capripoxvirus* genus (1). LSDV is mainly transmitted by blood-sucking arthropods (2–5). While LSD manifests with high morbidity, its mortality and fatality rate remain relatively low. The occurrence of LSD outbreaks has a negative impact on the well-being and productivity of infected cattle. This includes a reduction in body weight and a decline in milk yield. Additionally, it impacts the financial resilience of farmers, and at country level it has adverse consequences on international trade (6–8). It is estimated that LSD outbreaks in Asia caused an economic burden of approximately USD 1.45 billion (9). However, there are still limited comprehensive studies conducted at the national level in Asia.

The first LSD outbreak in Asia was reported in Bangladesh in 2019, after which LSDV spread to various countries in South, East, and Southeast Asia. The affected countries include China, Nepal, Vietnam, Myanmar, Malaysia, Hong Kong, Laos, and Taiwan (10–18). In 2021, Thailand experienced LSD outbreaks that were reported throughout the country. This situation is deemed to have had a significant adverse impact on the cattle industry (19).

In response to the nationwide LSD outbreaks, the Department of Livestock Development (DLD), a division of the Ministry of Agriculture and Cooperative, implemented a comprehensive set of preventive and control measures. These measures, initiated immediately after the first confirmed LSD outbreak in Thailand, included: [1] Restricting cattle movements to prevent disease spread; [2] Closing live cattle markets to minimize animal contact; [3] Implementing vector control strategies through insecticide use; and [4] Launching farmer awareness campaigns to educate about LSD clinical signs and disease transmission routes. These initial interventions aimed to contain the outbreak, reduce disease transmission, and increase vigilance among cattle farmers across the country (19).

Following the initial interventions, livestock authorities launched comprehensive vaccination campaigns across the country. These campaigns aimed to immunize a large portion of the cattle population against LSD, providing broader protection and helping to control the spread of the disease (20).

Northern Thailand, particularly Chiang Mai and Lamphun Provinces, is a focus of premium milk production, and has a high density of dairy farms. The region’s first LSD outbreak was documented in Lamphun in June 2021, which subsequently spread to Chiang Mai’s dairy farms. Given that the susceptible population was naïve to LSDV, a significant number of dairy cattle were adversely affected, posing a substantial threat to dairy farmers. These outbreaks underwent thorough investigations by livestock authorities and received laboratory confirmations (21). Although the epidemiological characteristics and spread of LSDV has been studied in detail in Thailand (22–24), the financial losses of these LSD outbreaks remain under-researched. While prior studies have assessed the adverse outcomes of LSD outbreaks in northeastern Thai dairy farms, they primarily quantified the decline in bulk tank milk production, leaving other financial aspects largely unexplored (25).

Understanding the financial repercussions is important, as it provides the requisite information for making informed decisions, facilitates the effective allocation of resources, and guides the formulation of strategies to mitigate these losses efficiently.

This study aims to quantify the financial losses due to LSD outbreaks in dairy farms across two northern Thai regions.

## 2 Materials and methods

### 2.1 Study area and outbreak definitions

**Figure 1:**
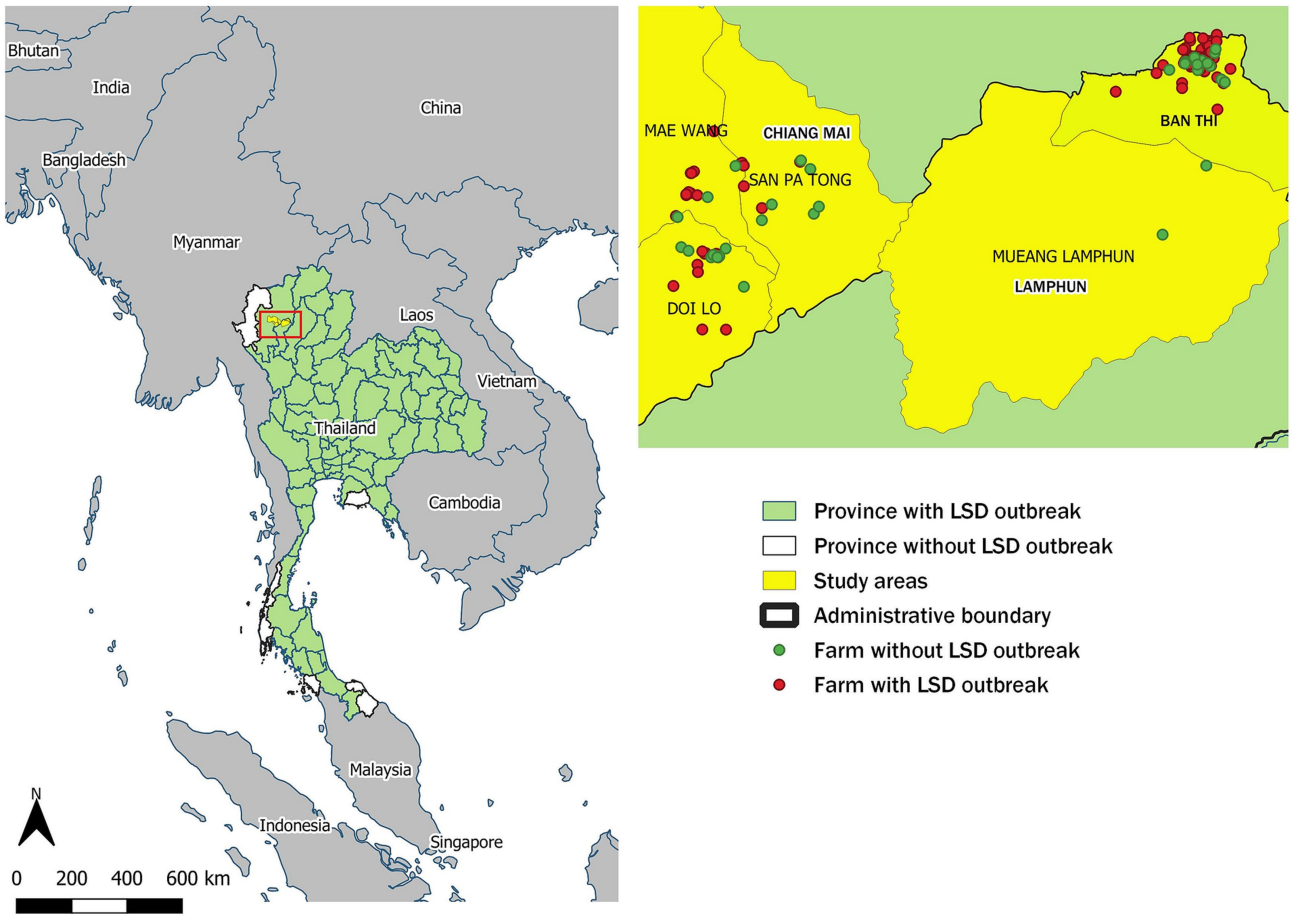
Geographic distribution of lumpy skin disease (LSD) outbreaks in Thailand. The left panel shows provinces in Thailand that reported LSD outbreaks, with the study area highlighted in yellow. On the right, green dots indicate the locations of non-LSD outbreak farms, while red dots pinpoint the locations of LSD outbreak farms.

This study was conducted in the intensive dairy farming areas of Chiang Mai and Lamphun provinces, where smallholder farms predominate, all of which are members of dairy cooperatives. The dairy farms involved in this study were affiliated with the Mae Wang Dairy Cooperative in Chiang Mai and the Lamphun Dairy Cooperative in Lamphun. Based on DLD reports (26), these cooperatives can be considered representative of other dairy cooperatives in the region, as dairy farms in other cooperatives share similar characteristics with those included in this study. These shared characteristics include cattle breeds, herd sizes, overall farm management practices, and a history of no previous LSD outbreaks. The geographical distribution of these farms is depicted in Figure 1. The LSD outbreaks within these farms received official confirmation from the DLD. Rigorous outbreak investigations were initiated to identify cattle manifesting clinical signs of LSD across all farms within the designated study regions. To confirm the presence of LSD, veterinary officials collected blood and tissue samples from a representative subset of cattle within the affected dairy farms. These samples underwent PCR testing to confirm the diagnosis of LSD (20). Furthermore, during the post-outbreak phase livestock keepers and DLD representatives met to assess the effectiveness of control measures, financial impacts and lessons learned.

In this study, in alignment with prior studies, an LSD outbreak farm is defined as farms having at least one cattle showing clinical signs consistent with the disease. These signs include raised, circular, firm nodules on the skin or mucosal surfaces, ranging in diameter from 1 to 5 cm as well as enlargement of superficial lymph nodes or swelling of a limb or lower body (27–29).

### 2.2 Questionnaire surveys

From June to December 2021, a comprehensive questionnaire survey was conducted across two dairy farm categories: those with animal(s) with LSD clinical signs (outbreak farms) and those without animals with clinical signs (non-LSD outbreak farms). This survey commenced after a three-month lull in new LSD cases, marking the post-outbreak phase.

The survey covered all dairy farms belonging to the Lamphun and Mae Wang dairy cooperatives, totaling to 89 and 44 farms, respectively. These two cooperatives were included because of their good record keeping and the willingness of farmers to take part of the study. A veterinary from DLD administered the survey using a standardized questionnaire, as detailed in a previous study (22). The questionnaire comprised three sections: farm demographics, epidemiological data, and farm financial information (Table 1). As part of the data recording system under the Good Agricultural Practice program implemented by the DLD, all farmers are required to report monthly herd population data, including the number of calves, heifers, lactating cows, and dry cows, to their respective dairy cooperatives. In addition to herd data, farmers must also provide financial information, such as expenses for purchased feed, farm equipment, disinfectants, veterinary services, and other related costs. The primary methods for recording this data include the use of logbooks and large whiteboards, which are standard across most farms (Supplementary Figures 1–3). Notably, approximately one-third of farmers have adopted mobile applications developed by DLD for data recording. Accordingly, during the interview, farmers were encouraged to refer to data from farm logbooks or other records, such as whiteboards, paper sheets, or notes stored on mobile phones.

**Table 1:** Components of the questionnaire and corresponding data utilized to assess the financial loss of lumpy skin disease outbreaks on dairy farms in northern Thailand.

Section	Data
Epidemiology data	Number of LSD case
	Number of LSD cases that die
	Date of LSD onset
	Date of most recent LSD case
Financial data	Selling price for milk
	Price of dead animal
	Vaccination expenditure
	Expenses from the treatment of LSD-infected cattle
	Expenses from antibiotics residual testing
	Expenses from disinfection
	Expenses from insect prevention and control

### 2.3 Ethical statement

Ethical approval for the study was obtained from the Faculty of Veterinary Medicine at Chiang Mai University, Thailand (Reference number: HS1/2565). A veterinarian conducted face-to-face interviews with the farmers, using the local regional language for communication. Prior to the interviews, verbal consent was obtained from all farmers through telephone communication, and they were asked to participate in the study. The interview took place only after the farmer provided their endorsement on a consent form.

### 2.4 Data analysis

#### 2.4.1 Epidemiological characteristics

Four measures of disease were used in the analysis: morbidity rate, mortality rate, case fatality rate, and herd attack rate. All of these rates were calculated on a farm level basis and expressed as a percentage. Morbidity rate was determined as the total number of animals that showed clinical signs of LSD divided by the total cattle population in the farm during the outbreak investigation (30). The mortality rate was estimated as the number of cattle that showed clinical signs of LSD and then died divided by the total number of cattle on the farm (30, 31). Case fatality rate was determined as the number of cattle that showed clinical signs of LSD and then died divided by the number of cattle that exhibited LSD signs (30, 32, 33). Further, the average and standard deviation of the morbidity rate, mortality rate and case fatality rate were determined. The herd attack rate was computed by dividing the number of farms with an LSD outbreak by the total farms in the study area (34).

The end of the outbreak was defined as the date on which the last farm in the study reported a confirmed LSD outbreak. This was verified with the affected farm, the head of the dairy cooperative, and the livestock veterinarian that no new outbreaks occurred after this final confirmation.

#### 2.4.2 Estimation of production losses and additional costs

**Figure 2:**
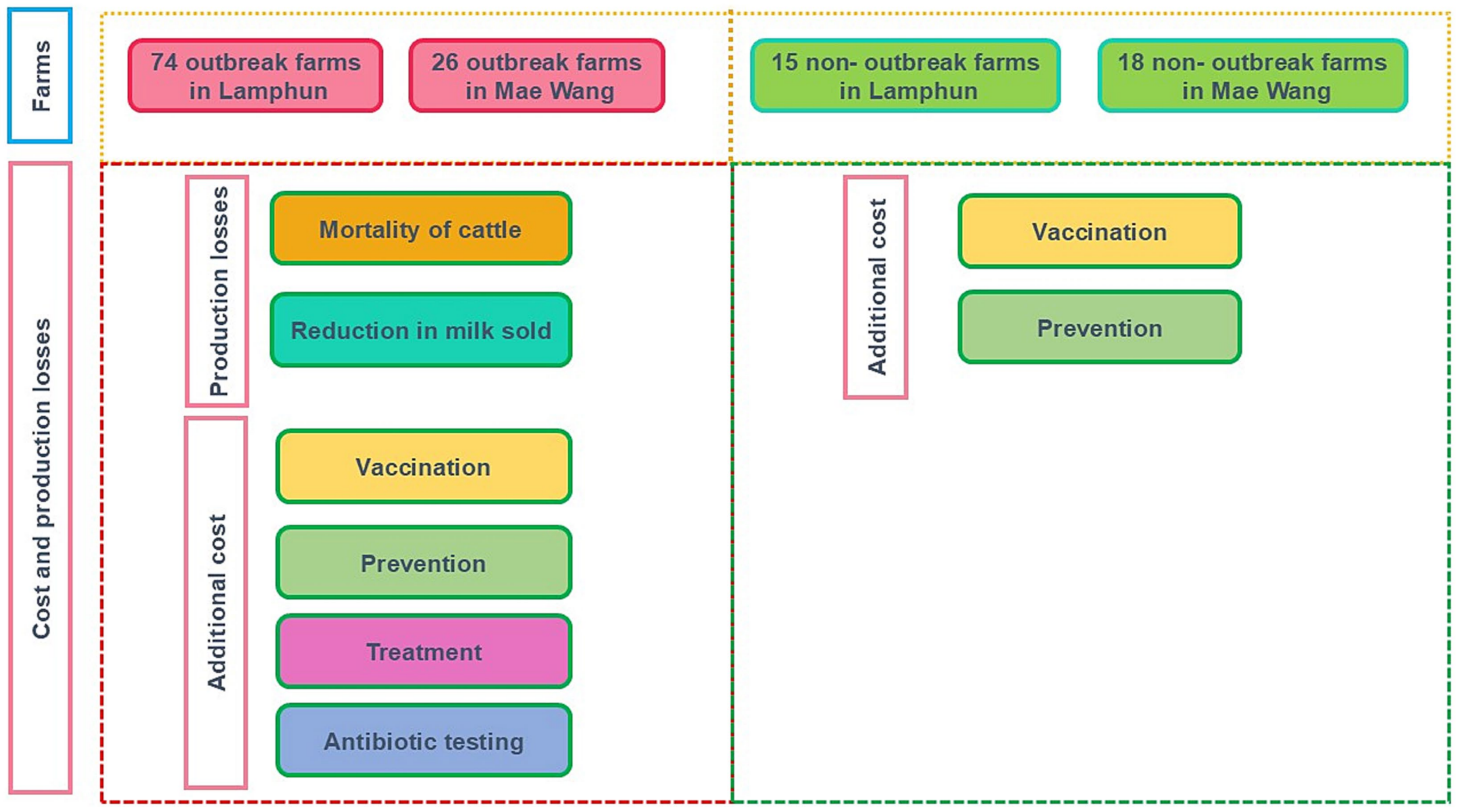
Number of farms in each study areas. Costs and losses considered for farms with and without lumpy skin disease outbreak are also illustrated.

Figure 2 illustrates the number of LSD outbreak farms and non-LSD outbreak farms, along with production losses and additional costs considered in the present study. Calculations were done separately for LSD outbreak and non-LSD outbreak farms.

##### 2.4.2.1 Production losses

###### 2.4.2.1.1 Mortality loss

The mortality loss $\left(\mathrm{Mortalit}y_{\mathrm{Loss}}\right)$ refers to the financial loss from animals affected by LSD that subsequently died. The loss is calculated as the product of the number of affected animals that died and the price at which an affected animal would have been sold. The mortality loss is calculated by the following equation adopted from previous studies (8, 29, 35, 36):


$$\mathrm{Mortalit}y_{\mathrm{Loss}}=n*\mathrm{pric}e_{\mathrm{animal}}$$

where, n is the number of cattle that died because of LSD infection. Price refers to the selling price of an animal, based on weight of animals, condition (e.g., healthy or sick) and local market price.

###### 2.4.2.1.2 Reduction of milk sold

The reduction of milk sold $\left(\mathrm{Mil}k_{\mathrm{Loss}}\right)$ pertains to the diminished revenue farmers encounter due to a decline in the volume of milk sold due to LSD. The loss can be attributed to a decline in milk production from the disease or the need to discard milk during the antibiotic withdrawal period for treating secondary infections from LSD. The reduction of milk sold is calculated by the following equation modified from previous studies (8, 29, 35, 36):


$$\mathrm{Mil}k_{\mathrm{Loss}}=\mathrm{YieldLoss}*\mathrm{pric}e_{\mathrm{milk}}$$

where YieldLoss is the quantity of the total milk losses from lactating cows affected with LSD in kilograms recorded by the farmer and price refer to milk price (per kilogram).

##### 2.4.2.2 Additional costs

###### 2.4.2.2.1 Treatment cost

Treatment cost $\left(\mathrm{Treatmen}t_{\mathrm{Cost}}\right)$ denotes the expenditure incurred by farmers for the treatment of LSD-affected animals, including veterinary service fees, antibiotics used for secondary infections, Non-Steroidal Anti-Inflammatory Drugs (NSAIDs), vitamins, minerals, and assorted supportive treatments. The treatment cost is calculated by the following equation adapted from previous studies (8, 29, 35, 36):


$$\begin{array}{l} \mathrm{Treatmen}t_{\mathrm{Cost}}=\mathrm{Vet}\;\mathrm{Fees}+\mathrm{Antibiotics}+\mathrm{NSAIDs}+\\ \mathrm{Vitamins}+\mathrm{Supportive} \end{array}$$

where, VetFees represent the fee of veterinary service cost to visit the farm and treat LSD affected animals during the LSD outbreak in the farm. Antibiotic is the expenditure of antibiotic medicine used both in systemic and tropical used. NSAIDs is the cost of Non-Steroidal Anti-Inflammatory Drugs applied in affected animals. Vitamins is the cost of vitamins and minerals used during the outbreak in affected animals. Supportive refer to the expenses that cover supportive treatment affected animal such as intravenous administration solutions and wound dressing.

###### 2.4.2.2.2 Vaccination cost

Vaccination cost ( $Vacc_{\mathrm{Cost}}$ ) represents the total expenditure borne by farmers for procuring the LSD vaccine, inclusive of the costs associated with its administration. This cost was incurred as part of the farm’s disease prevention and control measures. Within the study area, only commercially available LSD vaccines, procured directly by the farmers, were utilized. The farmers paid the entire cost of the vaccine without any subsidies. The vaccination cost is estimated by the following equation modified from previous studies (29, 35, 36):


$$Vacc_{\mathrm{Cost}}=n*\mathrm{pric}e_{\mathrm{vaccine}}$$

where n is the total number of vaccinated animals on the farm and price is the cost of vaccination per animal including administration cost. Livestock authority performed vaccination.

###### 2.4.2.2.3 Prevention cost

Prevention cost $\left(\mathrm{Preventio}n_{\mathrm{Cost}}\right)$ represents the aggregate expenditure incurred by farmers on measures implemented for LSD control and prevention, excluding vaccination costs. These measures include a variety of interventions across farms, such as installing insect nets in housing areas, using anti-insect lighting lamps, applying insecticides and disinfectants, and improving farm environments by maintaining cleanliness and removing potential insect habitats, such as dung. These interventions were particularly emphasized by DLD and adopted during the period when the disease was prevalent on the farms. The prevention cost compute by the following equation modified from previous studies (29, 35, 36):


$$\mathrm{Preventio}n_{\mathrm{Cost}}=Net+\mathrm{Lamp}+\mathrm{Insecticide}+\mathrm{Disinfectant}+\mathrm{Envi}$$

where Net corresponds to the cost associated with the installation of insect nets, Lamp represents the expenditure on anti-insect lighting lamps, Insecticide denotes the cost of insecticide agents, Disinfectant signifies the expenditure on disinfectant agents and, Envi is the payment for improving the farm environment, such as administration cost of farming waste and manure removal from the farm as well as decrease insect vectors habitat.

###### 2.4.2.2.4 Antibiotics residual testing cost

Antibiotics residual testing cost $\left(\mathrm{AB}O_{\mathrm{Cost}}\right)$ is the expenditure associated with laboratory testing for antibiotic residues in milk to ensure the safety and marketability of milk from lactating cows affected by LSD that underwent antibiotic treatment. This testing is crucial to ascertain that the milk produced is free from antibiotic residues before it is sold (37). Antibiotics residual testing cost is calculated using the following equation.


$$\mathrm{AB}O_{\mathrm{Cost}}=n*\mathrm{pric}e_{\mathrm{testing}}$$

where *n* is the total number of milk samples tested from the farm during the LSD outbreak and *price* is the cost of testing for antibiotic residues per milk sample.

##### 2.4.2.3 Total financial impact

The total financial losses $\left(\mathrm{TotalLosses}\right)$ for the LSD outbreak farms were computed using the following formula modified from previous studies (8, 29, 35, 36):


$$\begin{array}{l} \mathrm{TotalLosses}=Vacc_{\mathrm{Cost}}+\mathrm{Preventio}n_{\mathrm{Cost}}+\mathrm{Treatmen}t_{\mathrm{Cost}}+\\ \mathrm{AB}O_{\mathrm{Cost}}+\mathrm{Mortalit}y_{\mathrm{Loss}}+\mathrm{Mil}k_{\mathrm{Loss}} \end{array}$$

For non-LSD outbreak farms, the total cost (8, 29, 35, 36) was determined as follow:


$$\mathrm{TotalCost}=Vacc_{\mathrm{Cost}}+\mathrm{Preventio}n_{\mathrm{Cost}}$$

All financial data were initially gathered in Thai Baht (THB). For broader interpretation, these values were subsequently converted to US Dollars (USD). The exchange rate applied was 1 USD equivalent to 32.72 THB, which was the average rate prevailing from June to September 2021, as reported by the Bank of Thailand (38).

A heatmap was created to illustrate the total financial loss and its components for each farm and to visualize the differences between LSD outbreak farms and non-LSD outbreak farms.

The differences in means of total financial losses between LSD outbreak farms and non-LSD outbreak farms were analyzed using Generalized Linear Model (GLM). Initially, total financial loss $\left(y_{i}\right)$ was specified as the dependent variable, with herd status (LSD outbreak or non-LSD outbreak farms) included as an independent variable. Herd size was incorporated as covariates to account for potential confounding effects in the model. However, the residuals $\left(\varepsilon_{i}\right)$ from the model did not meet the normality assumption. To resolve this, a natural log-transformation was applied to the total financial loss data $\log\left(y_{i}\right)$ . Further, the residuals from the GLM with log-transformed data were subsequently assessed to check assumptions about normality and homogeneity of variance assumptions. Normality was evaluated using a normal quantile plot and the Shapiro–Wilk test, while homogeneity of variance was checked by examining the residuals plotted against the fitted values.

In addition, a GLM was applied to analyze the differences in mean milk production between LSD outbreak farms and non-LSD outbreak farms, focusing on data from the one-month period preceding the outbreak. Furthermore, the average milk production over the first 3 months during the outbreak period was compared between LSD-outbreak farms and non-LSD outbreak farms. For all statistical analyses, the level of statistical significance was set at *α* = 0.05.

### 2.5 Software

R version 4.3.3^1^ and the “*tidyverse*” package were used for data management, analysis, and “*ggplot2*” package was used for graph creation. The lm function from R was utilized for GLM. Maps were generated using the open-source QGIS software, version 3.34.^2^

## 3 Results

### 3.1 Characteristics and features of dairy farms

The study included 133 dairy farms, collectively housing 7,543 dairy cattle across two dairy cooperatives. These herds consisted mainly of cross-bred Holstein-Friesian cattle. Overall, the median herd size was 54 cattle, with individual farm herd sizes ranging between 12 and 175 animals. Based on data from 3 months prior to the outbreak, the studied farms produced an average daily milk production of 291.46 ± 171.62 kg (mean ± standard deviation), with a range of 9–916 kg. The standard selling rate for the raw milk stood at 0.57 ± 0.01 USD per kilogram, underscoring that raw milk sales were the principal revenue stream for these dairy farmers. The herd size and milk production characterized by study area and herd status are shown in Table 2.

**Table 2:** Farm characteristics of farms with and without LSD outbreaks stratified by dairy cooperative.

	Herd size (animals head)		Milk production (kg/day)	
	Mean ± SD	Median (min–max)	Mean ± SD	Median (min–max)
Lamphun dairy cooperative				
Non-LSD outbreak farms (*n* = 15)	41.00 ± 24.40	31 (14–106)	175.30 ± 132.25	110 (23–455)
LSD outbreak farms (*n* = 74)	57.50 ± 32.00	55 (12–175)	284.10 ± 166.88	270 (50–965)
Mae Wang dairy cooperative				
Non-LSD outbreak farms (*n* = 18)	54.27 ± 21.14	55 (18–89)	259.11 ± 119.60	202 (100–570)
LSD outbreak farms (*n* = 26)	65.23 ± 30.97	57 (24–163)	303.00 ± 147.73	302 (67–640)
Overall	56.70 ± 30.20	54 (12–175)	272.14 ± 156.80	251 (23–965)

Furthermore, analysis of milk production data from 1 month prior to the LSD outbreaks revealed that the average daily milk production on non-LSD outbreak farms was 236.81 ± 133.57 kg, which was lower than the 309.32 ± 179.32 kg observed on LSD outbreak farms (*p* < 0.05). Additionally, the results showed that daily milk production over the first 3 month of the outbreak was significantly higher in LSD-outbreak farms which was 289.01 ± 161.60 kg compared to non-LSD outbreak farms which was 221.02 ± 130.57 kg, *p* < 0.05.

Only one of the 133 dairy farms included in the study had no history of LSD vaccination, and this farm experienced an LSD outbreak. On the farms that had vaccinated, the owners independently purchased and administered homologous LSD vaccines. These vaccinations were conducted after the first LSD outbreak was reported in each study area.

### 3.2 Epidemiological characteristics

**Figure 3:**
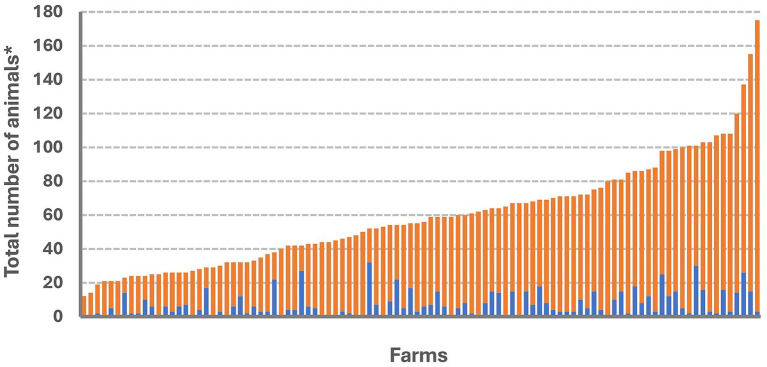
Number of cattle with and without clinical signs of lumpy skin disease (LSD). For each farm, the blue color bar represents the total number of cattle with clinical signs of LSD while the orange color bar depicts the number of cattle without clinical signs of LSD. *Animals including with the groups of lactating, dry, pregnant cow, heifer, calf, and ox.

The LSD outbreak spanned from June to December 2021. Out of the 133 farms studied, 100 farms had animals clinically affected (outbreak farms), giving a herd attack rate of 75.20%. The distribution of affected animals varied across farms, as illustrated in Figure 3. In LSD outbreak farms, the median (min–max) of morbidity rate, mortality rate and case fatality rate were 10.21% (1.25–64.30), 0% (0–12) and 0% (0–100) respectively. Other descriptive statistics of these rates including mean, standard deviation, minimum, maximum, quantiles and interquartile range are shown in Supplementary Table 1.

Cattle affected by LSD were observed across different groups, including lactating cows, dry cows, pregnant cows, heifers, calves, and oxen. Notably, calves exhibited the highest rates of morbidity (50.88%), mortality (62.20%), and case fatality (86.33%), as shown in Table 3. The farm that did not administer LSD vaccination experienced significantly higher rates of morbidity (64.30%), mortality (12%), and case fatality (18.50%) compared to farms that vaccinated against LSD. Additionally, it was observed that 99 farms with a history of LSD vaccination experienced LSD outbreaks.

**Table 3:** Morbidity, mortality, and case fatality rates due to LSD outbreaks in dairy farms within two dairy cooperatives, categorized by animal groups (*n* = 100).

	Number of affected farms	Mean ± SD	Median (min–max)	Interquartile range
Morbidity rate				
Lactating cow	66	18.42 ± 17.13	27.50 (8.33–100)	30
Dry cow	36	36.74 ± 24.80	0 (0–100)	20
Pregnant cow	47	41.17 ± 28.72	33.33 (4.55–100)	30
Heifer	50	26.81 ± 23.98	20 (3.70–100)	24.5
Calf	49	50.88 ± 31.30	50 (10–100)	50
Ox	16	10.00 ± 10.75	5.50 (1–33.33)	16.3
Mortality rate				
Lactating cow	5	4.22 ± 1.98	3.45 (2.94–7.70)	1
Dry cow	1	7.14	7.14	0
Pregnant cow	0	0	0	0
Heifer	7	8.10 ± 3.27	7.14 (3.03–12.50)	4.1
Calf	10	62.20 ± 41.54	81.66 (11.11–100)	83.33
Ox	1	20	20	0
Case fatality rate				
Lactating cow	5	23.00 ± 6.50	20 (16.67–33.33)	5
Dry cow	1	33.33	33.33	0
Pregnant cow	0	0	0	0
Heifer	7	42.06 ± 41.00	33.33 (6.66–100)	56.11
Calf	10	86.33 ± 24.67	100 (33.33–100)	15
Ox	1	100	100	0

### 3.3 Financial impact

#### 3.3.1 LSD outbreak farms

**Figure 4:**
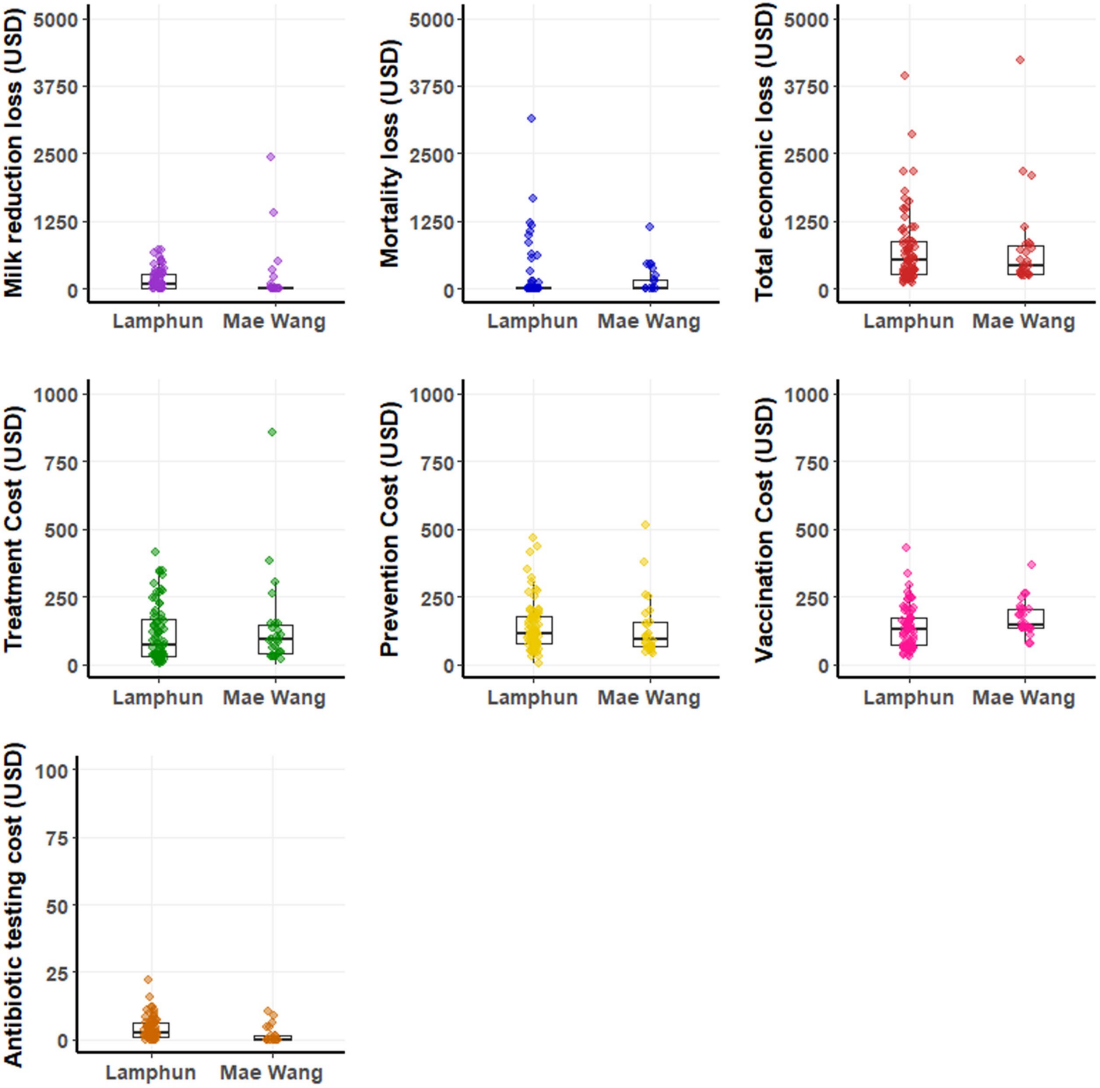
Boxplots depicting production losses and additional costs on dairy farms impacted by lumpy skin disease outbreaks within the Lamphun and Mae Wang dairy cooperatives, located in Lamphun and Chiang Mai provinces, northern Thailand.

The average financial loss was 727.38 ± 720 USD per LSD outbreak farm (ranging from 101 to 4,217 USD). This loss mainly came from cattle mortality and reduction in milk sold. Extra costs related to treatment, prevention, and vaccination also had important contribution to the financial burden (Figure 4 and Table 4). In contrast, the costs for residual antibiotic testing were comparatively small, with a mean of 5 ± 4.10 USD per farm varied from 1 to 22 USD (Table 4). A graphical depiction of these financial impacts and variation across farms, stratified by dairy cooperative, is presented in Figure 4.

**Table 4:** Financial losses due to lumpy skin disease outbreaks in dairy farms within two dairy cooperatives (*n* = 100).

Losses	Number of farms	Mean ± SD	Median (min–max)
Mortality loss	23	700.94 ± 690.53	458 (92–3,148)
Income loss due to the reduction of milk sold	62	262.01 ± 368.37	142 (11–2,432)
Vaccination cost	99	145.41 ± 76.08	135 (29–428)
Prevention cost	100	140.08 ± 98.39	112 (5–513)
Treatment cost	97	120.00 ± 126.00	82 (3–856)
Antibiotics residual testing cost	66	5.00 ± 4.10	5 (1–22)
Total	100	727.38 ± 720.00	506 (101–4,217)

The data is presented in USD^a^ currency.

^a^1 USD = 32.72 THB (average between June and September 2021) (38).

Of the 100 farms affected by the LSD outbreaks, 23 had financial losses due to cattle fatalities, averaging a loss of 700.94 ± 690.53 USD per LSD outbreak farm (Table 4). Milk from cattle infected with LSD and subsequently treated with antibiotics was typically withheld for a period of 6 days. This led to a loss of income for 62 farms, attributed to the unsold milk from lactating cows undergoing antibiotic treatment. The financial losses in this context varied between 11 USD and 2,432 USD, with a mean loss of 262.01 ± 368.37 USD per farm (Table 4). The mean expenditure for treatment stood at 120 ± 126 USD per farm, while the mean vaccination costs averaged was 145.41 ± 76.08 USD (ranging from 29–428 USD), depending on the cattle population of each farm. It is noteworthy that all participating dairy farms had adopted preventive strategies, encompassing measures like insect vector management and rigorous housing sanitation protocols. The mean expenditure for these preventive actions during the LSD outbreak was estimated at 140.08 ± 98.39 USD for each farm. Details of financial costs and losses are presented in Supplementary Tables 2, 4.

#### 3.3.2 Non-LSD outbreak farms

**Figure 5:**
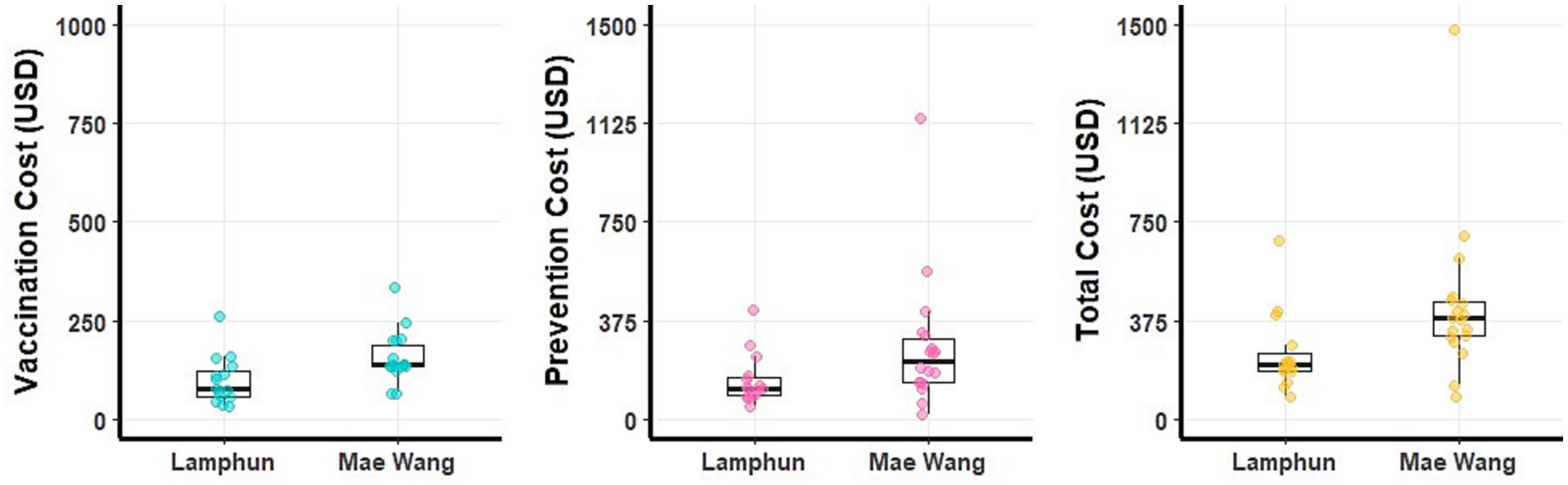
Boxplots illustrating the costs on non-LSD outbreak farms within the Lamphun and Mae Wang dairy cooperatives, located in Lamphun and Chiang Mai provinces, northern Thailand.

At the farm level, non-LSD outbreaks farms reported an average additional cost of 349.19 ± 257.01 USD (ranging from 83 to 1,482 USD), with vaccination expenses averaging 130.66 ± 67.02 USD (ranging from 34 to 336 USD) and preventive measures costing 218.53 ± 205.20 USD on average (Table 5). A detailed breakdown of these costs for individual farms, stratified by dairy cooperative, is illustrated in Figure 5. Additional details of financial costs are presented in Supplementary Tables 3, 5.

**Table 5:** Financial losses due to lumpy skin disease (LSD) outbreaks in dairy farms within two dairy cooperatives without LSD outbreaks (*n* = 33).

Losses	Mean ± SD	Median (min–max)
Vaccination cost	130.66 ± 67.02	135 (34–336)
Prevention cost	218.53 ± 205.20	150 (16–1,146)
Total	349.19 ± 257.01	313 (83–1,482)

The data is presented in USD^a^ currency.

^a^1 USD = 32.72 THB (average between June and September 2021) (38).

#### 3.3.3 Comparison of financial losses between LSD outbreak farms and non-LSD outbreak farms

**Figure 6:**
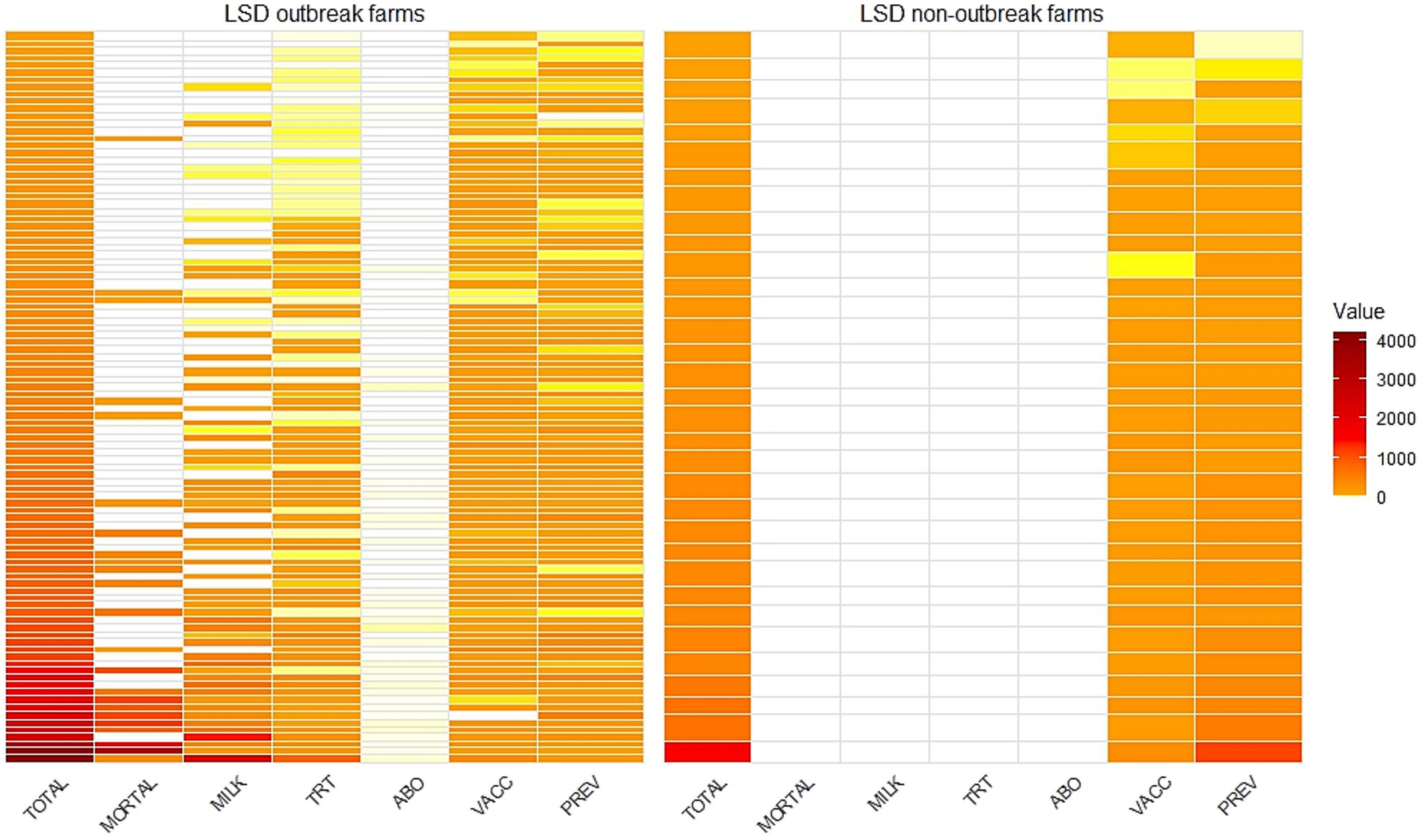
A heatmap illustrating the total economic losses (TOTAL) categorized by mortality loss (MORT), reduction in milk sales (MILK), treatment costs (TRT), antibiotic residue testing costs (ABO), vaccination costs (VACC) and prevention costs (PREV) stratified by farms with lumpy skin disease outbreaks (LSD outbreak farms) and farms without outbreaks (non-LSD outbreak farms). Values are in USD.

The heatmap provides a visual representation of the financial losses incurred by individual farms due to LSD (Figure 6). It clearly highlights that both LSD outbreak farms and non-LSD outbreak farms incurred costs for vaccination and prevention measures. However, only LSD outbreak farms faced additional financial losses from cattle mortality, reduced milk production, LSD treatment, and antibiotic testing.

Base on the GLM analysis, the financial losses incurred by LSD-outbreak farms were significantly higher than those of non-LSD outbreak farms (Table 6).

**Table 6:** Comparison of total financial losses between farms with lumpy skin outbreaks (LSD-outbreak farms) and farms without lumpy skin disease outbreaks (non-LSD outbreak farms).

Group	Total financial losses (mean ± SD)
LSD outbreak farms	727.38 ± 720.00^a*^
Non-LSD outbreak farms	349.19 ± 257.01^b^

*Different superscripts indicate statistical significance (*p* < 0.05).

## 4 Discussion

This study provides a thorough assessment of the financial losses due to LSD outbreaks on dairy farms, encompassing both LSD outbreak and non-LSD outbreak farms across two different areas in northern Thailand. It represents the first analysis in the country specifically addressing the negative financial impact of LSD outbreaks on dairy herds.

The primary financial losses for LSD outbreak farms were due to cattle mortality, especially in calves, and reduced milk production. These losses varied across farms, influenced by factors such as the number of affected cattle, case fatality rates, and volume of milk losses. This aligns with previous studies that identified mortality and reduced milk production as the main contributors to economic losses during outbreaks in Kenya and Ethiopia (29, 35), though a study in Bangladesh suggested that treatment costs were the major source of losses (39). In dairy cattle, the mortality rates of calves and heifers are significant (36). Mortality among dairy cattle not only results in a direct financial loss from the animal’s intrinsic value but also causes a decline in revenue due to diminished milk production and decreasing herd size. Cattle that survive LSD often exhibit a reduction in milk yield, leading to decreased sales for a farm over a long time period. Our analysis indicates that the quantity of milk sold is inversely proportional to the number of LSD-affected lactating cows undergoing antibiotic treatment and the duration of such treatment. This observation is consistent with previous studies, which have documented a marked decrease in milk production in LSD outbreak farms, especially when analyzing monthly production metrics before, during, and after outbreak periods (25). Furthermore, it should be noted that the average milk yield on non-LSD outbreak farms was lower than that on LSD outbreak farms when examining the outbreak data. A similar trend was observed when analyzing milk production data from 1 month prior to the outbreaks. The difference in milk production between outbreak and non-outbreak farms may be attributed to factors such as the lactation stage of dairy cows (number of months post-calving), the proportion of high-producing cows within the herd, and management practices, including nutritional strategies, tailored to maximize milk production potential.

The treatment cost for LSD-affected cattle varied across farms due to differing treatment practices among dairy farmers. The combination of using (or not using) antibiotics, nonsteroidal anti-inflammatory drugs, vitamin supplements, and other supplements led to variations in overall treatment expenses. These costs differed from the findings of the studies done in Kenya and Ethiopia (29, 36), likely due to variations in treatment protocols, prevention and control costs, production losses and the different variables in the total loss calculation. The total financial loss experienced by LSD outbreak farms in this study closely align with the reported 755 USD for exotic breed cattle in Kenya (29). However, these losses were lower than those documented in Ethiopia, where the estimated loss per LSD outbreak herd was 1,176 USD (35). When compared to the Balkan countries, the findings of this study indicate much lower financial losses. In 2016, the average cost per outbreak herd in the Balkans was 869 EUR (approximately 955.90 USD) in Albania, 6,994 EUR (approximately 7,693.40 USD) in Bulgaria, and 3,071 EUR (approximately 3,378.10 USD) in the Former Yugoslav Republic of Macedonia (40). The differences in findings between this study and those from other regions can be attributed to various factors, including cattle breed, age, immunity status, farming systems (e.g., intensive versus extensive) and control measures implemented at country level (e.g., stamping out). For instance, studies in East Africa included both intensive and extensive farming systems, whereas this study focused solely on small-scale farms. Additionally, the dairy cattle in this study were all crossbred Holstein Friesians (>75% HF), which differ from the cattle breeds examined in other settings.

Typically, in areas affected by LSD outbreaks in Thailand, veterinary authorities recommended preventive measures for all farms, including vaccination, the use of disinfectant on farms and insect vector control (22). The dairy farmers in these study areas adhered to these recommendations. All of them used insecticides and disinfectants, incurring costs that varied among farms due to factors such as the frequency of insecticide application and the duration of use. Additionally, 132 out of 133 farms used live attenuated homologous LSD vaccines, which were administered by veterinarians; however, LSD cases were reported in 100 farms. This may be due to instances where vaccinations were administered shortly before or during the actual LSD outbreak, therefore not giving animals enough time to develop an immune response and protection before exposure. Moreover, factors such as vaccine handling, storage, maintenance of the cold chain, vaccination methods, and the use of unauthorized vaccines (27) could contribute to incomplete herd protection. We recommend continuing the vaccination program to ensure that animals maintain sufficient immunity to the disease, using high-quality vaccines handled properly. Furthermore, the average vaccination cost per herd in our study was higher than those reported in other studies. For instance, a study conducted in Balkan countries in 2016, which used live homologous vaccines, reported vaccination costs were 8.2 EUR (approximately 9.02 USD), 22.5 EUR (approximately 24.75 USD), and 37.2 EUR (approximately 41 USD) per farm in Albania, Bulgaria, and the Former Yugoslav Republic of Macedonia, respectively (40). Additionally, a study in Kenya estimated vaccination costs against LSD at 11 USD per farm for exotic breeds and 2 USD per farm for indigenous breeds (29). The variation in vaccination costs observed between this study and others is likely due to differences in herd sizes, vaccine cost to the farmers and level of subsidies, and administration costs across countries.

In this study, the herd attack rate for LSD was high, reaching up to 75%, which might be attributed to insufficient herd immunity in several herds. Morbidity and mortality rates at within farms though were lower than those observed in other dairy herds in Thailand (23) and naïve beef cattle herds in northeastern Thailand (22, 41). The lower morbidity rate may be attributed to farmers vaccinating their dairy cattle. However, the overall case fatality rate in this study matches those reported in other studies in Thailand (22, 23, 41, 42). The highest morbidity, mortality, and case fatality rates were observed in calves. This aligns with other studies (8, 43, 44) which suggest that younger animals are more likely to die from the disease. Moreover, according to the farm investigation and data traced back with the area’s veterinary authority from the DLD, no cases of abortion were reported. Several other studies conducted in Thailand did not identify abortion as a prominent occurrence (19, 22).

Previous research in Thailand evaluating the impact of LSD has predominantly focused on losses related to milk production (25), leaving a gap in comprehensive data on other associated costs and financial losses. This study addresses this critical knowledge gap by providing a broader analysis of the financial losses at the farm level due to LSD outbreaks in Thailand. It also contributes to the limited body of work on this topic from Asian countries more broadly. We recommend further studies across other Asian nations and production systems to offer a more comprehensive regional understanding of the financial losses due to LSD at farm, national and regional levels.

This study had a number of limitations, including potential recall bias among farmers and the absence of data on the long-term consequences of outbreaks. Recall bias is a common challenge in studies reliant on questionnaire surveys. Given that farmers in this study demonstrated a well-established recording system, the chances of recall bias is reduced. However, data related to the duration of LSD-induced illnesses, milk reduction and the associated costs of disease prevention measures were not systematically collected and farmers had to provide some estimates of these. This might have resulted in financial losses being either exaggerated or underestimated, depending on the data provided by the farmers. Moreover, it should be noted that subclinical cases may lead to underestimation, as this study defines LSD-affected animals based on clinical signs. However, the vast majority of herds were naïve, and cattle infected with LSDV would likely show clinical signs of the disease. Furthermore, this study did not explore the potential long-term consequences of LSD outbreaks, including the possible negative effects on the reproductive capabilities of affected cattle, weight loss, secondary infection (e.g., mastitis) and the financial impact of acquiring replacements for lost livestock or changes in herd size. Future studies would benefit from employing more advanced economic modeling, exploring the long-term effects of LSD on farms and assessing broader socio-economic impacts incorporating value chain analyses.

## 5 Conclusion

This study estimates the financial losses resulting from LSD outbreaks, in farms with and without LSD clinical cases, in two dairy farming areas. In farms with LSD-cases, these losses primarily stem from cattle mortality and the costs associated with implementing prevention and control measures. Both farms outbreak and non-outbreak by LSD incurred expenses for prevention tools and practices. The present study is the first to report financial losses due to LSD in Thailand providing valuable insights that enhance our understanding of the negative impacts of LSD outbreaks in dairy cattle.

## Data Availability

The original contributions presented in the study are included in the article/Supplementary material, further inquiries can be directed to the corresponding author.

## Footnotes

^1^ www.r-project.org

^2^ www.qgis.org
