# Supplementary material for: An evaluation of financial losses due to lumpy skin disease outbreaks in dairy farms of northern Thailand
Source: Front Vet Sci. 2025 Jan 24;11:1501460. doi: 10.3389/fvets.2024.1501460 (PMC11804113; doi:10.3389/fvets.2024.1501460)
Supplement: Supplementary file 1 [file Supplementary_file_1.docx]

Supplementary Material

**Supplementary Table 1.** Morbidity, mortality and case fatality rate caused by lumpy skin disease in dairy farms.

|  | **Mean ± SD** | **Median (min-max)** | **Quantile 1** | **Quantile 3** | **Interquartile Range** |
| --- | --- | --- | --- | --- | --- |
| Morbidity rate | 14.03 ± 13.91 | 10.21 (1.25 - 64.30) | 4.23 | 18.81 | 14.58 |
| Mortality rate | 0.86 ± 2.20 | 0 (0 - 12) | 0 | 0 | 0 |
| Case fatality rate | 8.90 ± 24.57 | 0 (0 - 100) | 0 | 0 | 0 |

**Supplementary Table 2.** Financial losses due to lumpy skin disease outbreaks in dairy farms within Lamphun dairy cooperative (n=74). The data is presented in USD^a^ currency.

| **Losses** | **Number of Farms** | **Mean ± SD** | **Median (min-max)** |
| --- | --- | --- | --- |
| Mortality loss | 15 | 847.59 ± 796.33 | 641 (92 - 3,148) |
| Income loss due to the reduction of milk sold | 55 | 204 .35± 189.77 | 135 (11 - 721) |
| Vaccination cost | 74 | 136.75 ± 78.19 | 132 (29 - 428) |
| Prevention cost | 74 | 142.35± 94.01 | 115 (5 - 468) |
| Treatment cost | 72 | 113.46 ± 104.88 | 75 (3 - 414) |
| Antibiotics residual testing cost | 58 | 4.99 ± 4.18 | 4 (1 - 22) |
| Total | 74 | 717.11 ± 666.20 | 527 (101 - 3,926) |

^a^ 1 USD = 32.72 THB (average between June to September 2021, Bank of Thailand, 2021)

**Supplementary Table 3.** Financial losses in dairy farms without lumpy skin disease outbreaks within Lamphun dairy cooperative (n=15). The data is presented in USD^a^ currency.

| **Losses** | **Mean ± SD** | **Median (min-max)** |
| --- | --- | --- |
| Vaccination cost | 99.59 ± 59.78 | 78 (34 - 260) |
| Prevention cost | 149 .20 ± 95.93 | 119 (51 - 419) |
| Total | 248.79 ± 148.33 | 208 (88 - 678) |

^a^ 1 USD = 32.72 THB (average between June to September 2021, Bank of Thailand, 2021)

**Supplementary Table 4.** Financial losses due to lumpy skin disease outbreaks in dairy farms within Mae Wang dairy cooperative (n=26). The data is presented in USD^a^ currency.

| **Losses** | **Number of Farms** | **Mean ± SD** | **Median (min-max)** |
| --- | --- | --- | --- |
| Mortality loss | 8 | 425.96 ± 312.79 | 405 (153 - 1,131) |
| Income loss due to the reduction of milk sold | 7 | 715.03 ± 886.30 | 353 (31 - 2,432) |
| Vaccination cost | 25 | 171.02 ± 64.22 | 153 (76 - 367) |
| Prevention cost | 26 | 133.60± 111.68 | 94 (40 - 513) |
| Treatment cost | 25 | 138.79 ± 174.49 | 92 (18 - 856) |
| Antibiotics residual testing cost | 8 | 4.96 ± 3.53 | 5 (1.5 - 11) |
| Total | 26 | 756.60 ± 869.15 | 423 (232-4,217) |

^a^ 1 USD = 32.72 THB (average between June to September 2021, Bank of Thailand, 2021)

**Supplementary Table 5.** Financial losses in dairy farms member of Mae Wang dairy cooperative, without lumpy skin disease outbreaks (n = 18). The data is presented in USD^a^ currency.

| **Losses** | **Mean ± SD** | **Median (min-max)** |
| --- | --- | --- |
| Vaccination cost | 156.55 ± 62.84 | 138 (67 - 336) |
| Prevention cost | 276.32 ± 252.79 | 223 (16 - 1,146) |
| Total | 432.86 ± 299.85 | 384 (83 - 1,482) |

^a^ 1 USD = 32.72 THB (average between June to September 2021, Bank of Thailand, 2021)
