# Supplementary material for: An evaluation of financial losses due to lumpy skin disease outbreaks in dairy farms of northern Thailand
Source: Front Vet Sci. 2025 Jan 24;11:1501460. doi: 10.3389/fvets.2024.1501460 (PMC11804113; doi:10.3389/fvets.2024.1501460)
Supplement: Supplementary file 2 [file Supplementary_file_2.docx]

**Supplementary Figure 1 to 3**


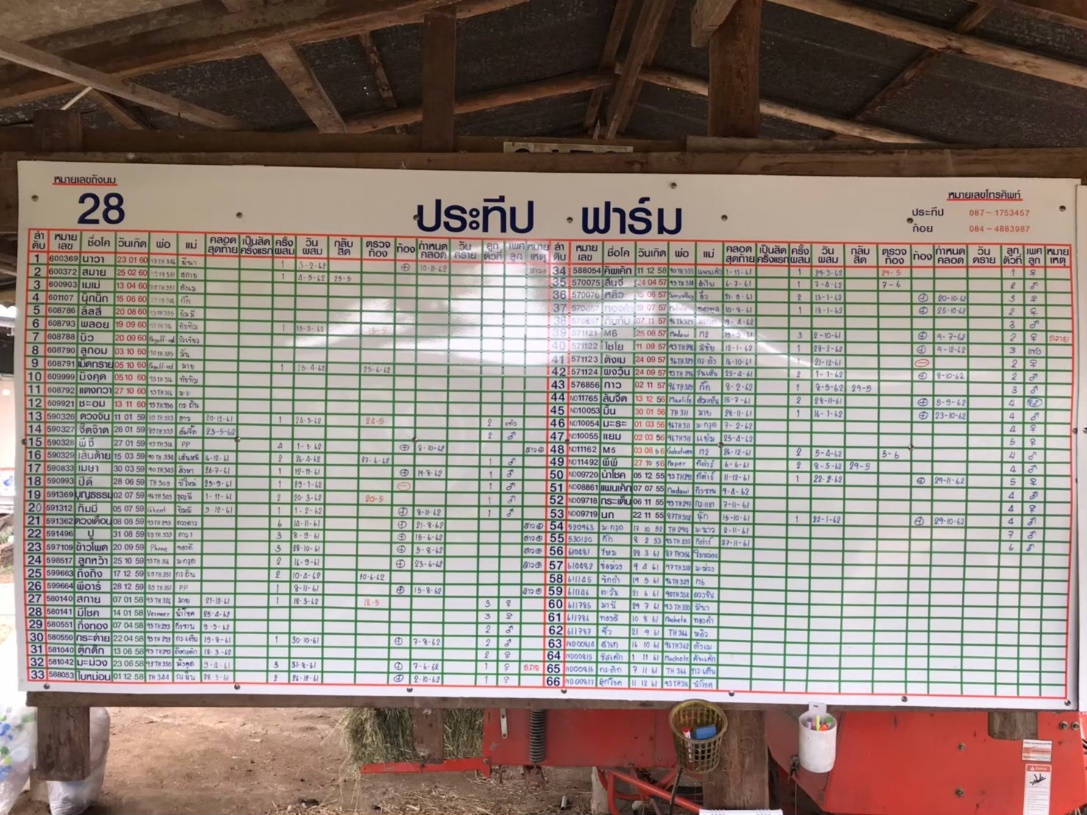


Supplementary Figure 1. Example of farm data recording using whiteboards.


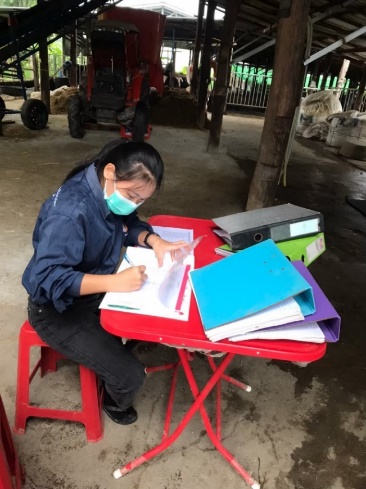

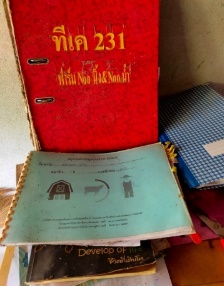

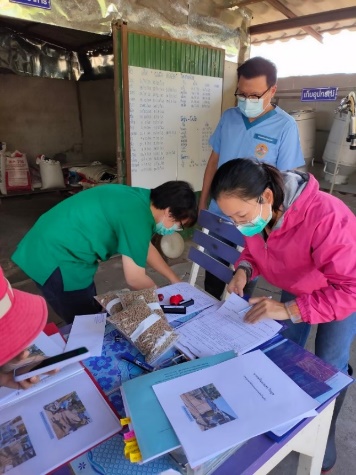


Supplementary Figure 2. Examples of farm data recording using logbooks, paper sheets, and stored notes.


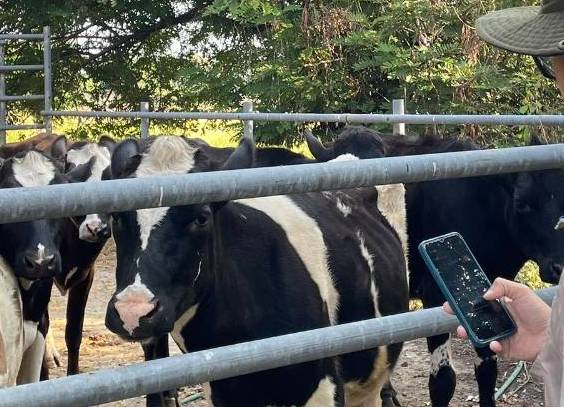


Supplementary Figure 3. Example of farm data recording using open-source mobile applications. Details about the application can be accessed via the following link: https://biotech.dld.go.th/webnew/images/Information/iFarmer/M-iFarmer.pdf.
